# Case report: A case of recurrent cervical cancer with bronchial and esophageal metastases presenting with hemoptysis and dysphagia

**DOI:** 10.3389/fonc.2024.1375035

**Published:** 2024-04-19

**Authors:** Xiao Yu, Shixiang Dong, Wenjie Wang, Xin Sun, Yankui Wang, Fengsheng Yu

**Affiliations:** ^1^ Department of Gynecology, The Affiliated Hospital of Qingdao University, Qingdao, China; ^2^ Medical College, Qingdao University, Qingdao, China

**Keywords:** recurrent cervical cancer, bronchial metastasis, esophageal metastasis, immune checkpoint inhibitor, tumor-associated dermatomyositis

## Abstract

**Background:**

The treatment outcomes and prognosis for recurrent cervical cancer are generally poor, with a 5-year survival rate of only 10%–20%.

**Case presentation:**

In this case, the patient is a young woman who experienced a recurrence 5 years after the initial treatment of cervical cancer. Her primary symptoms were hemoptysis and dysphagia, indicative of hilar and mediastinal lymph node metastases, with further involvement of the bronchus and esophagus. Additionally, the patient also presented with tumor-associated dermatomyositis. Following combined treatment with albumin-bound paclitaxel, carboplatin, bevacizumab, and cadonilimab, the patient’s tumor was effectively controlled.

## Case presentation

A 39-year-old young woman was hospitalized in December 2016 due to persistent vaginal bleeding for 1 month. The admission diagnosis was human papillomavirus (HPV)-related cervical cancer (IB1, G2), according to the International Federation of Gynecology and Obstetrics (FIGO) 2009. The patient underwent laparoscopic radical hysterectomy, bilateral salpingectomy, pelvic lymphadenectomy, and bilateral ovarian transposition. Postoperative pathology revealed moderately differentiated squamous cell carcinoma of the cervix, with deep one-third of the cervical stroma infiltrated, lymphovascular space invasion (LVSI) positive, and no pelvic lymph node metastasis. According to the Sedlis criteria, pelvic external radiation therapy along with concurrent chemoradiotherapy was performed. The patient’s postoperative follow-up showed no abnormalities in 57 months.

In September 2021, the patient developed irregular edematous erythema on the trunk and limbs, with visible vesicles and ruptures. She consulted the Rheumatology and Immunology Department and was diagnosed with dermatomyositis (DM). After symptomatic treatments, the patient’s DM was stably controlled, followed by oral administration of prednisone acetate, 10 mg daily, for maintenance therapy. Meanwhile, she returned to the outpatient clinic for follow-up, and no recurrence of cervical cancer was detected.

**Figure 1:**
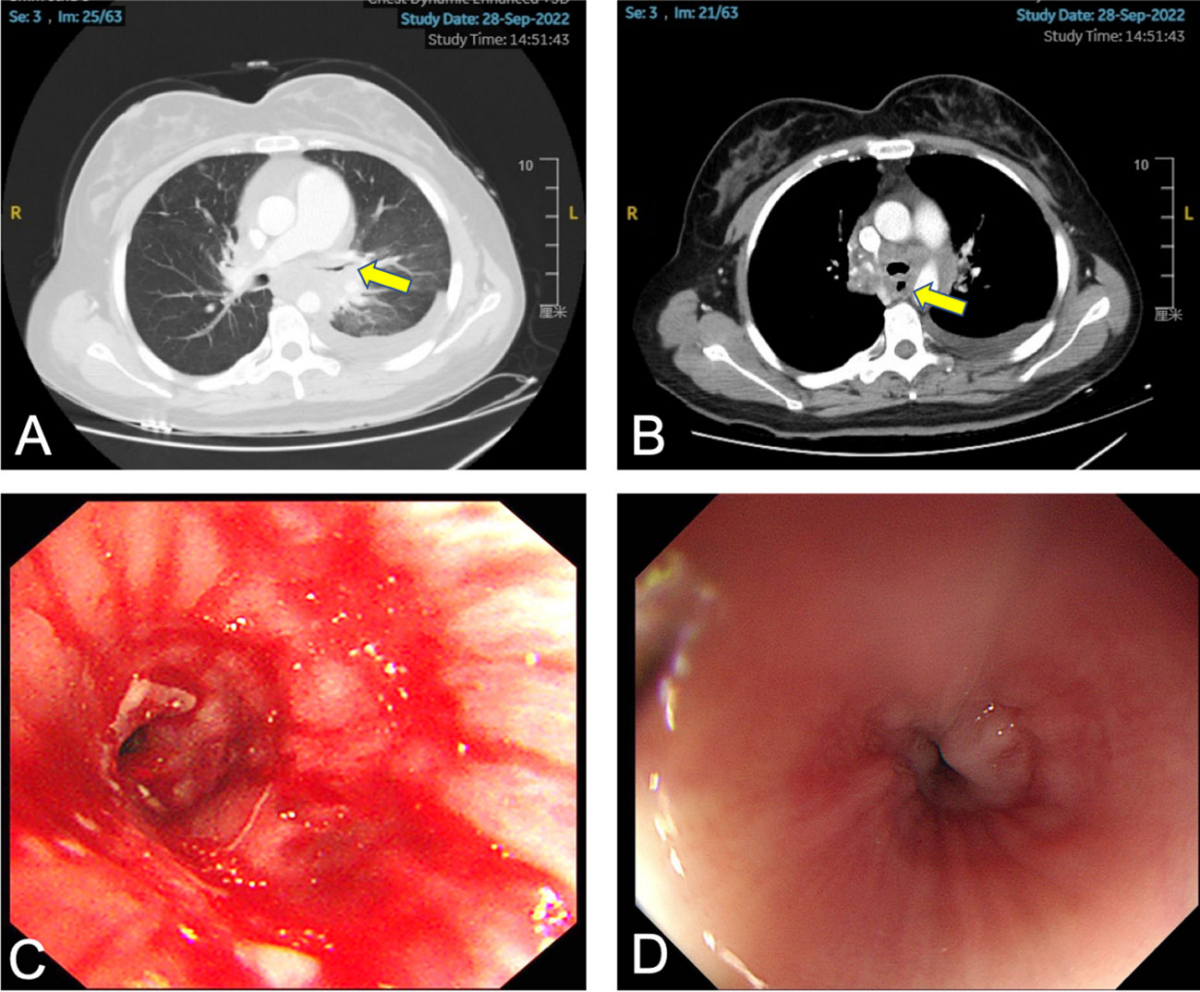
Patient’s chest CT, bronchoscopy, and gastroscopy examination. **(A)** The arrow indicates the narrowed left main bronchus. **(B)** The arrow indicates the narrowed esophagus. **(C)** Bronchoscopy shows narrowing of the left main bronchus with white necrotic material attached. **(D)** Gastroscopy shows circumferential narrowing of the esophagus with smooth mucosa.

**Figure 2:**
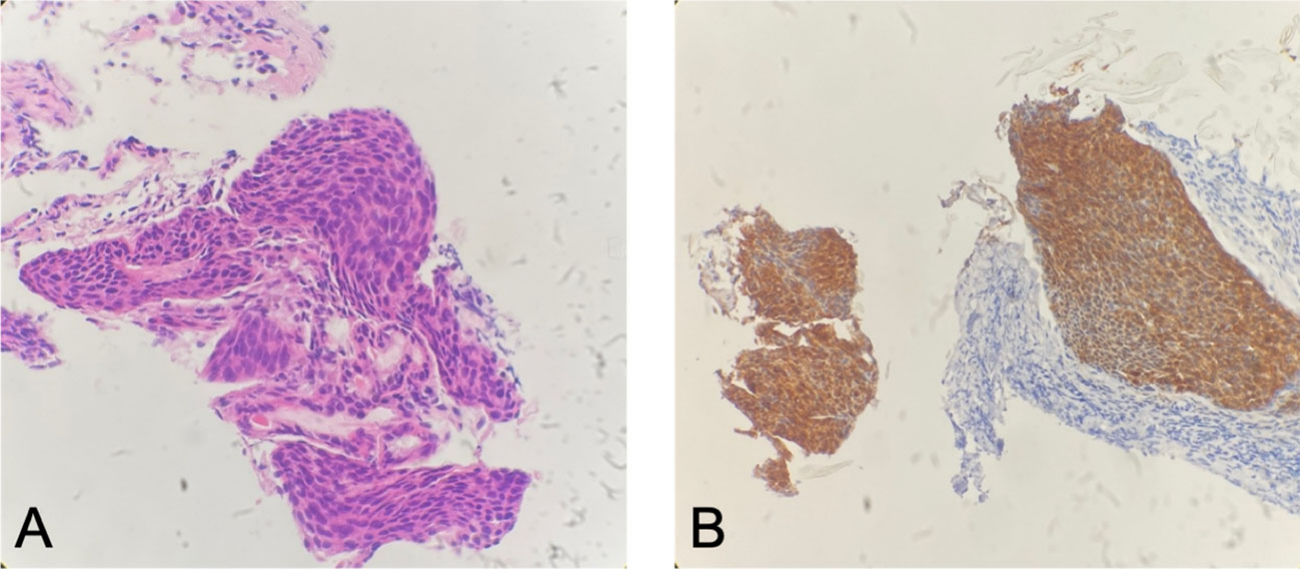
Patient’s bronchial biopsy pathology. **(A)** Bronchial biopsy pathology (H&E staining, ×100). **(B)** Strong positive expression of P16 (immunohistochemistry staining, ×100).

**Figure 3:**
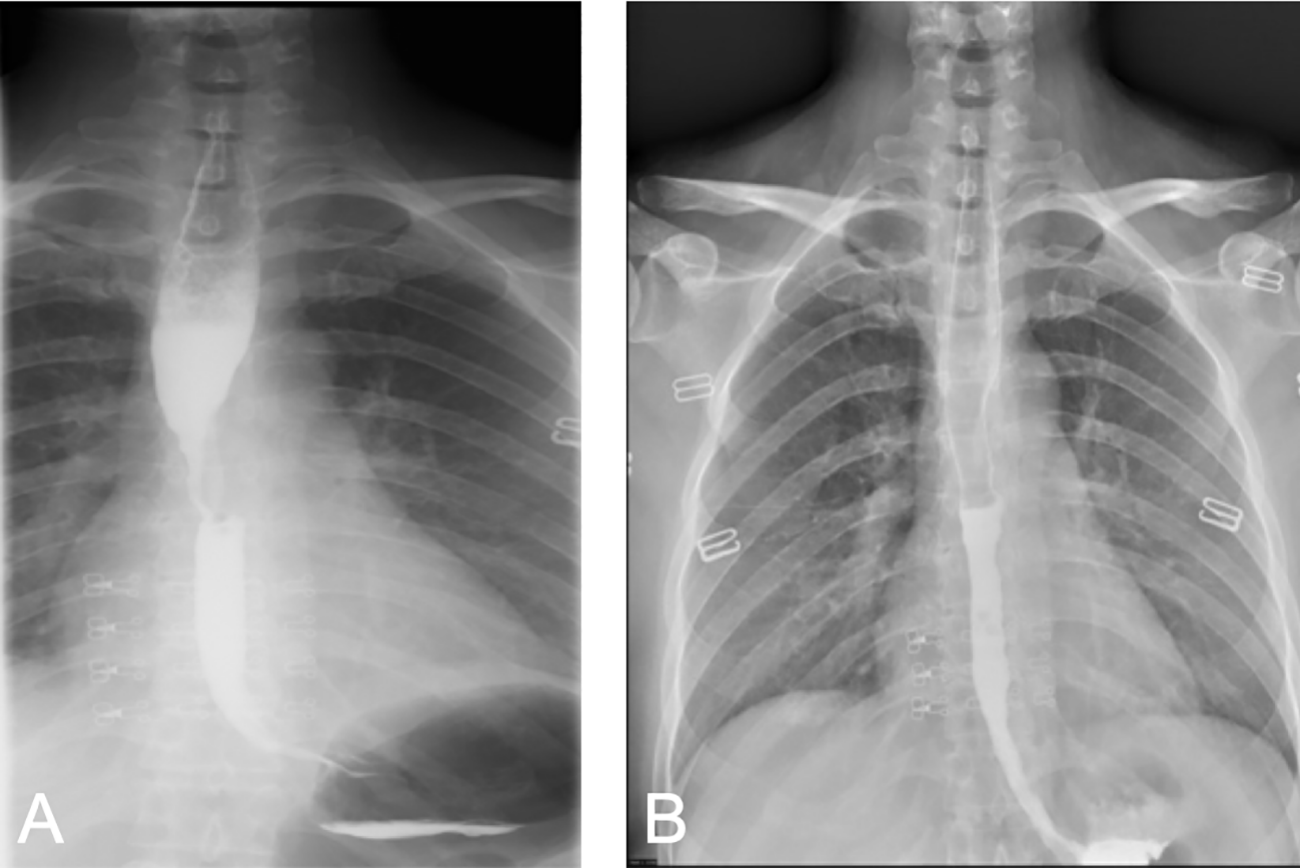
Comparison of barium swallow radiography before and after treatment. **(A)** Before treatment, localized narrowing of the esophageal lumen, involving an area of approximately 38 mm, with dilation above the narrowed segment. **(B)** After treatment, the local esophageal wall mucosa appears slightly irregular.

In August 2022, the patient presented with symptoms of dysphagia, hoarseness, chest tightness, shortness of breath, coughing, and bloody sputum. Physical examination revealed red rashes and scattered vesicles on both upper arms, the back, and around both knee joints. Routine gynecological examination showed no abnormalities. Transcription intermediary factor 1-gamma (TIF1-γ) antibody was positive. Tumor marker screening indicated an elevated squamous cell carcinoma (SCC) antigen level of 45.06 ng/mL. Enhanced CT scans revealed partial stenosis and obstruction of the right upper lobe bronchus. Narrowing of the bilateral main bronchi and multiple enlarged lymph nodes in the hilum and mediastinum were observed. The middle segment of the esophagus (at the T5–T7 level) showed localized wall thickening with luminal stenosis and dilatation of the esophagus above (**Figures 1A, B**). Bronchoscopic examination revealed a narrowing of the openings of the bilateral main bronchi and a narrowing of the lumen of the left main bronchus with easily bleeding mucosa (**Figure 1C**). Pathological examination through bronchoscopy revealed squamous epithelial nests in the bronchial mucosa tissue. Based on morphology, immunohistochemistry results, and history, metastatic squamous cell carcinoma, likely originating from cervical cancer, was considered. Immunohistochemistry showed p16 (+), CK5/6 (+), p40 (partially +), and PD-L1-22C3 (tumor proportion score (TPS): −) (**Figure 2**). During gastroscopy, circumferential stenosis was observed at 30 cm from the incisors, preventing the passage of the endoscope, with the esophageal mucosa appearing smooth (**Figure 1D**). Barium meal examination showed localized esophageal luminal stenosis of approximately 38 mm, with dilation above the stenosis (**Figure 3A**). Based on these findings, recurrent cervical cancer with secondary malignant tumors in the bronchus, esophagus, and mediastinum accompanied by tumor-associated DM was confirmed.

**Figure 4:**
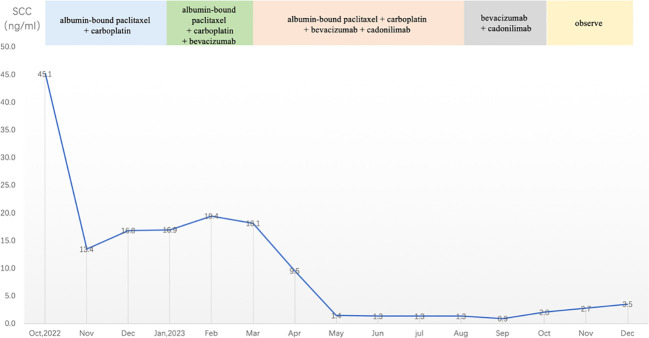
SCC antigen levels during treatment and observation (reference range 0–2.5 ng/mL). SCC, squamous cell carcinoma.

Following a multidisciplinary consultation involving the Departments of Gynecology, Oncology, Rheumatology and Immunology, and Radiology, the patient was considered to have an extra-pelvic recurrence of cervical cancer with concomitant tumor-associated DM. It was recommended to treat the DM while also initiating anti-tumor therapy. According to the 2022 cervical cancer National Comprehensive Cancer Network (NCCN) guidelines, the preferred systemic treatment for recurrent or metastatic cervical cancer is paclitaxel + cisplatin/carboplatin ± bevacizumab ± pembrolizumab (for PD-L1-positive patients). Given the patient’s recurrence of cervical cancer involving the trachea and esophagus, presenting with symptoms, like hemoptysis and dysphagia, and concurrent autoimmune disease DM, to reduce the use of steroid medication and to avoid gastrointestinal reactions caused by cisplatin, the patient was administered intravenous chemotherapy with albumin-bound paclitaxel (210 mg/m^2^) + carboplatin [area under the curve (AUC) = 5] every 3 weeks for a total of three cycles. After improvement in hemoptysis, bevacizumab (7.5 kg/m^2^) was added for two cycles. During chemotherapy, the patient’s SCC antigen levels initially decreased and then fluctuated and increased (**Figure 4**). After five cycles of chemotherapy, the radiological evaluation in February 2023 showed similarity to before chemotherapy treatment. Due to acute esophageal obstruction, gastroscopy was performed, confirming persistent esophageal stenosis.

**Figure 5:**
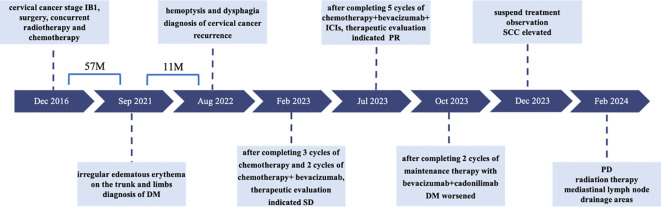
Treatment timeline for patient. DM, dermatomyositis; SD, stable disease; PR, partial response; SCC, squamous cell carcinoma antigen; PD, progressive disease.

After completing five cycles of chemotherapy, the patient’s therapeutic evaluation indicated stable disease (SD). Her DM remained stable. Following a multidisciplinary discussion, a decision was made to adjust the treatment plan to include “albumin-bound paclitaxel + carboplatin + bevacizumab (7.5 mg/kg) + cadonilimab (10 mg/kg)”, with the schedule set for five consecutive cycles (one cycle every 3 weeks). The patient’s hemoptysis, dysphagia, hoarseness, and other discomfort were completely relieved, and the SCC antigen levels decreased significantly. After undergoing 10 cycles of chemotherapy post-recurrence, radiological assessment in July 2023 showed apparent slight stenosis in the right upper lobe bronchus, a slight narrowing of the bilateral main bronchi, no significant enlargement of lymph nodes in the hilum and mediastinum, and local wall thickening in the mid-segment of the esophagus (at the T5–T7 level) with apparent slight stenosis in some pulmonary bronchi. Barium meal examination showed localized esophageal wall mucosa appears slightly irregular (**Figure 3B**). The patient’s tumor was well-controlled, and she was given maintenance therapy with bevacizumab (7.5 mg/kg) + cadonilimab (10 mg/kg). However, in October 2023, the patient’s DM worsened, leading to the cessation of maintenance therapy, and she was temporarily under observation. During this period, follow-up indicated an increase in SCC antigen levels from 1.98 ng/mL to 3.49 ng/mL, although radiological assessment did not reveal any tumorous lesions. A consultation with a radiation oncologist was required to consider radiation therapy to the mediastinal lymph node drainage areas. Treatment timeline for patient was shown in (**Figure 5**).

## Discussion

Approximately 30% of cervical cancer patients experience tumor recurrence or metastasis after initial treatment, with most recurrences occurring within 2 years post-treatment and fewer cases after 5 years (1). Recurrences can be categorized into intra-pelvic and extra-pelvic. Treatment for recurrent and metastatic cervical cancer is challenging, and prognosis is poor, with a 5-year survival rate of only 10%–20%, making it a difficult aspect of gynecologic oncology (2). The patient, in this case, a young woman, underwent surgery and concurrent chemoradiotherapy in 2016 for stage IB1 cervical cancer. She experienced a recurrence 5 years post-initial treatment, with no signs of pelvic recurrence. The presentation was an unusual extra-pelvic recurrence, primarily manifesting as hemoptysis and dysphagia, involving the bronchus and esophagus. The lung metastasis in this patient was not a parenchymal spread but involved the bronchial walls; the esophagus was also considered to be narrowed due to the tumor. The bronchial and esophageal changes were attributed to the invasion of surrounding tissues due to metastases to the pulmonary hilum and mediastinal lymph nodes. Literature indicates that the mediastinal metastasis rate for cervical cancer is approximately 1%, and with the continuous development of techniques such as PET-CT and endoscopic ultrasound-guided fine-needle aspiration, mediastinal metastasis can be more definitively diagnosed (3). While the incidence of lung metastases is approximately 20%–50%, bronchial metastases are rare, accounting for less than 5% (4). Distinguishing between primary and metastatic bronchial tumors in pathology morphology is challenging. In this case, the patient’s bronchial biopsy pathology revealed squamous cell carcinoma with a strong positive expression of P16 for immunohistochemistry staining. Considering the history, this was deemed a recurrence and metastasis of cervical cancer. The patient’s recurrence site was special and extensive, limiting radiation and surgical treatment. Considering the potential tissue fibrosis and difficult improvement in esophageal stenosis caused by radiation, the treatment choice was primarily systemic therapy.

The PD-L1 positivity rate in cervical cancer patients ranges from approximately 34.4% to 96% (5, 6), making immune checkpoint inhibitors (ICIs) a novel strategy for treating recurrent or metastatic cervical cancer. The 2022 cervical cancer NCCN guidelines list paclitaxel + cisplatin/carboplatin ± bevacizumab ± pembrolizumab (applicable for PD-L1-positive patients) as the preferred systemic treatment for recurrent or metastatic cervical cancer (7, 8). For this patient, who was PD-L1 negative and also suffering from tumor-associated DM, with initial symptoms of hemoptysis and dysphagia, we opted for a chemotherapy regimen of albumin-bound paclitaxel combined with carboplatin. After the patient’s hemoptysis symptoms improved, we added the anti-angiogenic drug bevacizumab. Following the initial chemotherapy, the patient’s tumor marker SCC antigen levels significantly decreased but fluctuated and increased. After completing five cycles of chemotherapy, the radiological assessment indicated SD. After multidisciplinary discussions and a thorough evaluation of the risks and benefits of using ICIs, we decided to add cadonilimab (a PD-1/CTLA-4 dual-target antibody). The AK104-201 study, a multicenter, open-label Phase 1b/II clinical trial conducted in China, included 111 patients with recurrent/metastatic cervical cancer who had previously received platinum-based chemotherapy but failed treatment. Of these, 100 patients were treated with cadonilimab and included in the efficacy analysis, showing an overall response rate (ORR) of 33%, with PD-L1-positive patients [combined positive score (CPS) ≥ 1] at 43.8% and PD-L1-negative patients at 16.7%. The study demonstrated that patients could benefit from cadonilimab treatment regardless of PD-L1 expression status (9, 10). The Chinese Society of Clinical Oncology (CSCO) guidelines recommend the use of cadonilimab in recurrent or metastatic cervical cancer, particularly for PD-L1-negative patients. In this case, we administered five cycles of cadonilimab treatment to the patient, resulting in significant improvement of the tumor and full relief of symptoms such as dysphagia and hoarseness, leading to the cessation of chemotherapy and entry into the maintenance treatment phase. However, as the patient’s DM worsened, we discontinued ICI treatment. During observation, SCC antigen levels gradually increased, but radiological examinations did not reveal positive findings. Five months after stopping chemotherapy, we decided to administer radiation therapy to the mediastinal drainage area.

Five years after the initial treatment, the patient was diagnosed with DM, and cervical cancer recurrence was confirmed 11 months after the onset of DM. The timing of DM and cancer recurrence was closely aligned. DM is an autoimmune disease primarily affecting the skin and skeletal muscles, with patients having a fivefold increased risk of concurrent malignancy compared to the general population, making it a paraneoplastic syndrome (11). TIF1-γ antibody is considered a serological marker for malignancy-associated adult DM, with TIF1-γ-positive DM patients having a 38% to 71% probability of concurrent malignancy (12). In this case, the patient was TIF1-γ positive, and her DM was exacerbated concurrent with the diagnosis of recurrent cervical cancer. The DM symptoms improved as the tumor was controlled, suggesting a tumor-associated DM. Given the patient’s PD-L1-negative status and concurrent autoimmune disease, the use of ICIs required special caution. However, no adverse symptoms emerged post-ICI administration in this patient. With the improvement in dysphagia, her dietary intake normalized, and her general health gradually improved, yielding positive treatment outcomes. Cancer patients with autoimmune diseases are not absolute contraindications for ICIs therapy, and potential risks and benefits must be weighed in clinical applications. According to the 2023 NCCN guidelines, autoimmune disease patients who have not received or only received low-dose immunosuppressive therapy and have controlled disease conditions may consider ICIs therapy (7). For this patient, we will closely monitor her condition and enhance early detection of adverse drug reactions to maximize therapeutic efficacy.

## Data availability statement

The raw data supporting the conclusions of this article will be made available by the authors, without undue reservation.

## Ethics statement

The studies involving humans were approved by the Ethics Committee of the Qingdao University Affiliated Hospital. The studies were conducted in accordance with the local legislation and institutional requirements. The participants provided their written informed consent to participate in this study. Written informed consent was obtained from the individual(s) for the publication of any potentially identifiable images or data included in this article.

## Author contributions

XY: Writing – original draft, Methodology, Visualization, Writing – review & editing. SD: Data curation, Writing – original draft. WW: Data curation, Writing – original draft. XS: Data curation, Writing – original draft. YW: Supervision, Writing – original draft. FY: Supervision, Writing – review & editing.
